# Endoscopic Color Doppler Ultrasonography for Esophagogastric Varices

**DOI:** 10.1155/2012/859213

**Published:** 2012-11-19

**Authors:** Takahiro Sato, Katsu Yamazaki

**Affiliations:** Department of Gastroenterology, Sapporo-Kosei General Hospital, Kita 3 Higashi 8, Chuo-Ku, Sapporo 060-0033, Japan

## Abstract

Esophagogastric varices are considered to be the most common complication in patients with portal hypertension. Endoscopic ultrasonography not only visualizes the surface of the varices but also provides detailed information about their internal structure. The direction of blood flow can be determined and its velocity measured only via endoscopic color Doppler ultrasonography (ECDUS). This can show graphically esophageal varices, paraesophageal veins, and passageways in esophageal variceal patients and gastric varices, perigastric collateral veins in gastric variceal patients. It is important to evaluate the hemodynamics of the portal venous system when treating the esophago-gastric varices. ECDUS is a useful modality for the evaluation of the detailed hemodynamics and the therapeutic effects of esophago-gastric varices.

## 1. Introduction

Esophagogastric varices are considered to be the most common complication in patients with portal hypertension. Endoscopic injection sclerotherapy (EIS) [1] and endoscopic variceal ligation (EVL) [2] are effective treatments for esophageal variceal bleeding. In Japan, there appears to be controversy in deciding which of the two is the best therapy for elective and prophylactic cases. Therefore, it is important to evaluate the hemodynamics of the portal venous system when determining the optimal choice of treatment for patients with portal hypertension. Recent technical advances have offered clinicians increasingly greater clarity in visualizing gastric varices. Gastric variceal bleeding is a common complication of portal hypertension and is associated with higher morbidity and mortality rates than hemorrhage from esophageal varices [3]. Bleeding gastric varices can be treated successfully by injection of cyanoacrylate. Balloon-occluded retrograde transvenous obliteration (B-RTO) is a new approach for the obliteration of collateral vessels connecting the portal venous system and systemic circulation, and has been evaluated recently for the treatment of gastric varices [4]. Gastric fundal varices associated with large gastro-renal shunt (GRS) [5] are a good indication for B-RTO, which has been performed widely in Japan [6–9]. In this paper, we review the hemodynamics of esophagogastric varices due to portal hypertension and describe the usefulness of endoscopic color Doppler ultrasonography (ECDUS). 

### 1.1. Hemodynamics of Esophageal Varices

Hepatofugal flow in the collateral veins (left gastric vein, short gastric vein, and posterior gastric vein) is involved in the formation of esophageal varices. The left gastric vein is the major site of esophageal varices in patients with portal hypertension. The morphological changes of the left gastric vein in portal hypertension have been elucidated by angiographic examinations [10–12]. It is generally thought that the blood flow in the left gastric vein may change from a hepatopetal direction to the hepatofugal direction in liver cirrhosis. Matsutani et al. reported that hepatofugal blood flow in the left gastric vein increased in direct correlation with enlargement of the size of the varices and a high flow velocity in the left gastric vein was strongly associated with variceal bleeding [13]. 

The palisade zone corresponds to the abdominal esophagus, beginning at the gastro-esophageal junction and extending superiorly for 4-5 cm [14]. The veins in this zone were distributed uniformly, in close proximity to each other and running parallel and longitudinally as a palisade. Palisade veins, which are normally seen in the lamina propria at the lower end of the esophagus, are called sudare like veins. The palisade veins run through the lamina propria and most end draining to the submucosal veins at the critical area. Noda stated that the ruptured veins were situated in the lamina propria and the rupture points were located near the area where the varicosed palisade veins are connected to the submucosal varices [15]. Arakawa and Kage revealed marked dilatation of the veins in the submucosa more often in patients with well-developed varices than in those without varices via the palisade zone, and they classified these cases into two groups: those with sudare like veins, and those with vascularity in which one or two large dilated vessels run through the submucosa [16]. Hashizume et al. classified the sudare-like veins as a palisading type and the dilated vessels as a bar type and reported that palisading type veins in the lamina propria were dilated introducing into the muscularis mucosae and were observed circumferentially observed in the submucosa [12, 17]. 

The routes of esophageal varices are mainly associated with gastric wall blood flow (left gastric vein, short gastric vein, and palisade vein), and perforating veins are recognized as additional passageways. There is little information in the literature on perforating veins [17–19]. 

### 1.2. Hemodynamics of Gastric Varices

The incidence of gastric varices has been widely reported, with Hosking and Johnson detecting gastric varices in 6% of patients with esophageal varices [20]. Sarin et al. detected gastric varices in 48 (16%) of 309 patients with cirrhosis, noncirrhotic portal fibrosis, or extrahepatic obstruction [21]. In contrast, Watanabe et al. reported the frequency of gastric varices in patients with portal hypertension to be 57% [5]. Gastric varices have been diagnosed by endoscopy, a useful modality for observing gastric varices of a certain size and extent. Endoscopy has a very sensitive predictive value for variceal hemorrhage [22]. However, there are few cases of red color-positive gastric varices and it is difficult to diagnose a high risk for bleeding of gastric varices. Still further, endoscopy is a limited modality for detecting gastric varices, given how deep the submucosal or extramural collateral veins of gastric varices are. 

The role of magnetic resonance and computed tomography (CT) imaging is limited in distinguishing between submucosal gastric varices and perigastric collateral veins, although both modalities allow assessment of the entire portal venous system [23, 24]. Balthazar et al. reported the usefulness of CT examinations for gastric varices and described a method for obtaining a detailed diagnosis in most patients with gastric varices [25]. In that study, overall findings of the portosystemic collateral channel in patients with portal hypertension could be obtained by CT scans. Willmann et al. demonstrated that multidetector row CT angiography is equivalent to EUS in the detection of fundal varices [26]. 

Transabdominal color Doppler ultrasonography is a useful for the diagnosis of gastric variceal hemodynamics and for evaluation of the therapeutic effects of gastric variceal treatment [27]. Color Doppler ultrasonography has advantages over other techniques in that it is a simple method, noninvasive, and can be used to calculate the blood flow velocity of gastric varices. In that study, color Doppler ultrasonography captured accurate images of venous flow in the gastric walls of 41 of 41 patients (100%). However, color Doppler ultrasonography requires a suitable acoustic window, and we found that color Doppler ultrasonography is limited in the detection of collateral veins in gastric variceal patients. Impediments such as bowel gas, body habitus, and cirrhosis can limit the value of sonography for assessing the portal venous system. 

 B-RTO has been evaluated recently for the treatment of gastric varices, and gastric fundal varices associated with large GRS are good indication for B-RTO. A high number of color flow images of GRS were produced using transabdominal color Doppler ultrasonography, which proved valuable when evaluating the hemodynamics of gastric variceal patients [27]. 

## 2. ECDUS 

Endoscopic ultrasonography (EUS) has become a very useful modality for the diagnosis of esophageal varices [28–30]. EUS not only visualizes the surface of the varices but also provides detailed information about their internal structure. ECDUS is better able to observe the detailed hemodynamics of esophageal varices better than conventional EUS. 

 Hemodynamic evaluation of the esophagogastric varices was performed by ECDUS using a PENTAX FG-36UX (forward-oblique viewing), 7.5 MHz, convex type, which provided 100 degree images (convex type ECDUS) or EG-3630UR (forward viewing), 10 MHz, electronic radial type, which provided 270 degree images (electronic radial-type ECDUS) (Pentax Optical, Tokyo, Japan). The HITACHI EUB565 or EUB8500 was used for the display (Hitachi Medical, Tokyo, Japan). 

 Exploration of esophagogastric varices was conducted by introducing deaerated water from an autoinfuser device into the stomach through the working channel. Velocities were assessed by the pulsed Doppler method, by positioning a sample volume of 1-2 mm in the center of the vessels. The color gain was adjusted so as to eliminate background noise, and the insonation angle was kept below 60 degree to minimize ambiguity in measurements of blood flow. To begin with, identification of esophagogastric varices was performed with B-mode scanning and then, color flow mapping was done. ECDUS is a method for detecting color flow images in blood vessels. The direction of blood flow and the measurement of velocity can be achieved only via ECDUS. 

 New electronic radial ECDUS was performed using a PENTAX EG-3670 URK (forward-view) with a distal tip diameter of 12 mm and a working length of 1250 mm. The range of tip reflection is 130 degree up and 60 degree down/right/left. A HITACHI EUB 7500, which provides 360 degree images, was used for display [31]. 

## 3. Hemodynamics of Esophageal Varices with ECDUS

ECDUS shows graphically esophageal varices, paraesophageal veins, and passageways. Sato et al. have reported previously on the usefulness of convex type ECDUS for evaluating the hemodynamics of esophageal varices [32–34]. 

**Figure 1:**
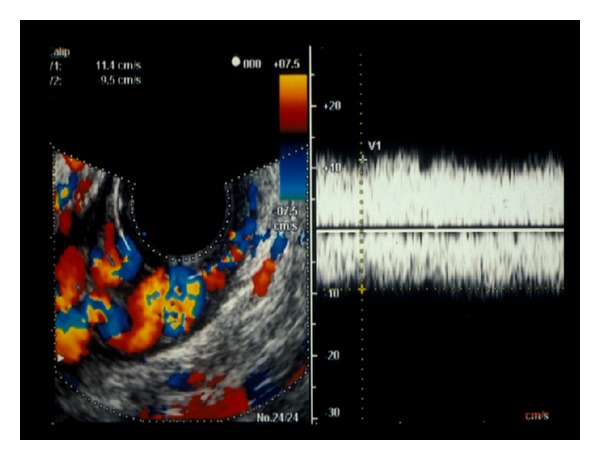
The image shows anterior branch dominant esophageal varices observed with endoscopic color Doppler ultrasonography.

 Hino et al. analyzed the morphology and hemodynamics of the left gastric vein using ECDUS to evaluate the development of esophageal varices [35]. They reported that hepatofugal blood flow velocity in the left gastric vein trunk increased with the size of the varices. The left gastric vein bifurcates into anterior and posterior branches. As the size of the varices enlarged, the branch pattern was more likely to be anterior branch dominant. EIS is recommended as the better choice of endoscopic treatments for the anterior branch dominant esophageal varices. The image shows anterior branch dominant esophageal varices observed with ECDUS (Figure 1). Vessel images of the palisade veins were discerned with ECDUS running parallel and longitudinally around the gastro-esophageal junction. However, the vessel images of palisade veins produced by ECDUS may show only part of the system of palisade veins. Observation of color flow images of palisade veins via ECDUS is difficult because of the fine vessels with low velocity [36]. Sato et al. reported the usefulness of an electronic radial ECDUS in evaluating the hemodynamics of esophageal varices in comparison with convex-type ECDUS, and color flow images of palisade veins were obtained in 12 of 26 (46.2%) cases with electronic radial ECDUS. In addition, the detection rate of palisade veins with electronic radial ECDUS was significantly higher than that with the convex-type ECDUS [37]. Endoscopic treatment is very safe and popular, but recurrence of varices is now becoming a serious problem. Intramucosal venous dilatation (IMVD) of esophageal varices has been observed frequently in follow-up endoscopy after endoscopic therapies [38]. IMVD has been evaluated as the regional tortuous dilatation of varices and indicates a risk of bleeding. The palisade veins remaining after endoscopic therapies are related to IMVD. 

**Figure 2:**
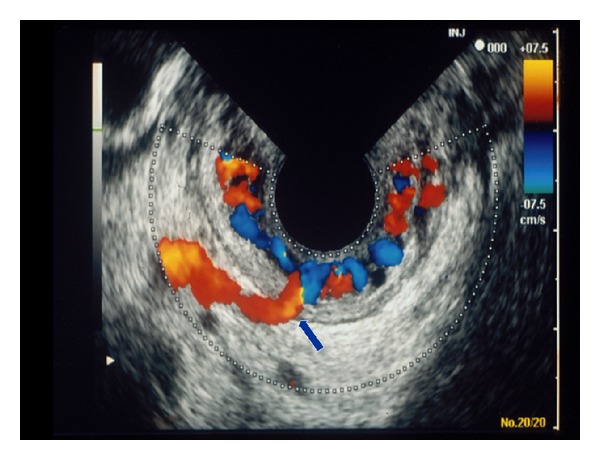
Inflow type perforating vein showed color flow image from the paraesophageal veins to the esophageal varices.

 Perforating veins are defined as communicating vessels between esophageal varices and paraesophageal veins. It is difficult to discern the perforating veins via CT scans or MR angiography. Perforating veins can be visualized via EUS, but the direction of blood flow in perforating veins cannot be determined by this method. The direction of blood flow in perforating veins can only be shown qualitatively by ECDUS [39]. Choudhuri et al. reported on perforating veins that connect the submucosal and paraesophageal collateral venous channels in the lower esophagus using EUS; these were observed in 15% of patients with small varices and 70% with large varices [29]. The perforating veins detected by ECDUS were classified into three types according to the flow direction. Type 1 showed inflow from the paraesophageal veins to the esophageal varices (Figure 2). Type 2 showed outflow from the esophageal varices to the paraesophageal veins. Type 3 was a mixed type that revealed both inflow and outflow [37]. In this paper, color flow images of perforating veins were obtained in 18 of 26 (69.2%) cases. The direction of blood flow in perforating veins is an important consideration in the therapeutic management of esophageal varices. Therefore, we should perform EIS on Type 1 for the purpose of obliterating esophageal varices and perforating veins. On the other hand, Type 2 is associated with diversion of esophageal variceal blood flow into the paraesophageal veins and is therefore equivalent to an extraesophageal shunt [40]. One must use great caution in performing EIS for Type 2 and Type 3 variceal patients and EIS should be performed at the anal site of outflowing perforating veins. Endoscopic variceal ligation (EVL) may be the optimum treatment for this type of varices [41]. 

Hemodynamic evaluation of portal hypertension reveals that hepatofugal flow in the collateral veins is involved in the formation of esophageal varices, and also that a hyperdynamic state from the lower esophagus to the cardiac area is involved in forming esophageal varices [42, 43]. The blood flow in the stomach wall supplied from the left gastric artery also participates in variceal blood flow in the early stage [44–46]. The hyperdynamic stage is caused by an increase in the arteriovenous anastomoses in the submucosal layer of esophagus and stomach by which the arterial blood flows in through the left gastric artery and proper esophageal artery. Observation of a pulsatile wave with convex type ECDUS is difficult because of the fine vessels. The detection rate of pulsatile wave with electronic radial ECDUS was significantly higher than using convex-type ECDUS. Electronic radial ECDUS provides extended 270 degree views (convex-type ECDUS provides 100 degree views), and this advantage provides clearer visualization of pulsatile waves in esophageal varices [37]. 

**Figure 3:**
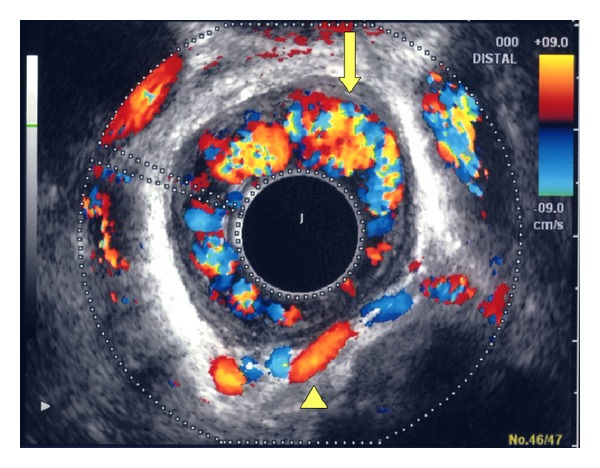
Vessel images of esophageal varices (arrow) and paraesophageal veins (arrowhead) were delineated clearly with new electronic radial endoscopic color Doppler ultrasonography.

Next, we show new electronic radial ECDUS images (Figure 3). Vessel images of esophageal varices and paraesophageal veins were delineated clearly in this case. We found two chief advantages over the old probe which seem to be that it is easier to manipulate in the distal esophagus than the old probe and that it produces 360 degree images instead of 60 or 270 degree images.

## 4. Hemodynamics of Gastric Varices with ECDUS

The introduction of EUS equipped with Doppler facilities has allowed the sonographic visualization of vessels and the evaluation of vascular blood flow along with morphology in the diagnosis of gastric varices [47, 48]. 

Iwase et al. reported that ECDUS is a useful method for evaluating gastric varices and endoscopic obliteration with cyanoacrylate glue [49]. 

Using ECDUS, the mean velocity of large and coil-shaped-type varices was found to be significantly higher than that of enlarged and tortuous-type varices. All four bleeding cases had velocities of >20 cm/s, and the velocities of the bleeding cases, were significantly higher than those of the nonbleeding cases [50]. Sato et al. assumed that ECDUS is superior to CT for measurement of blood flow velocity of gastric varices as well as wall thickness of submucosal gastric varices, suggesting that these determinations obtained by ECDUS are useful for the prediction of variceal bleeding. 

## 5. Hemodynamics of Esophageal Variceal Recurrence

Although endoscopic treatment is safe and popular, recurrence of esophageal varices has become a serious problem. EUS analysis of gastric cardiac vascular structures could be useful for predicting the recurrence of esophageal varices [51–53]. Two publications reported that severe, shallow-type paraesophageal vein and perforating vein detected using an EUS catheter probe after EIS correlated significantly with variceal recurrence [54, 55]. Other authors have reported previously that the development of a GRS reduces the frequency of variceal relapse [56, 57]. 

Ito et al. reported that the incidence of variceal relapse was lower in patients with nonvariceal systemic portal shunts than in patients without these shunts [58]. ECDUS can be used to evaluate the hemodynamic characteristics of esophageal varices before and after EIS and the data obtained can be used to predict the early recurrence of esophageal varices. 

In particular, detection of cardiac intramural vein and inflowing perforating vein by ECDUS after EIS showed a strong correlation with early variceal recurrence [59]. 

## 6. Therapeutic Effects of Gastric Varices

**Figure 4:**
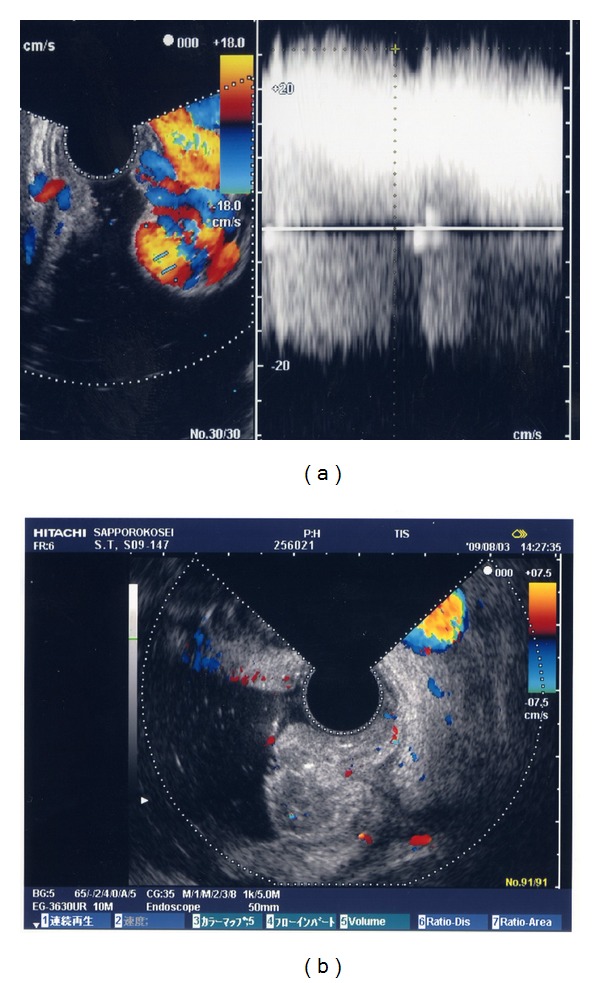
(a) Color flow images of gastric varices before treatment. (b) Endoscopic color Doppler ultrasonography shows the disappearance gastric variceal color flow images after endoscopic treatment with N-butyl-2-cyanoacrylate.

Bleeding gastric varices can be treated endoscopically by injection of cyanoacrylate. N-butyl-2-cyanoacrylate (Histoacryl, B.Braun Dexon GmbH Spangenberg, Germany) is a tissue glue monomer that instantly polymerizes and solidifies upon contact with blood. Use of N-butyl-2-cyanoacrylate for treating bleeding gastric varices, which generally confer a high mortality rate, was first reported by Soehendra et al. [60]. ECDUS is a very useful method to evaluate the therapeutic effect of endoscopic treatment with N-butyl-2-cyanoacrylate [61] (Figures 4(a) and 4(b)).

## 7. Gastric Varices Secondary to Splenic Vein Occlusion

Splenic vein occlusion (characterized by gastric varices and splenomegaly and with normal liver function) can result in left-sided portal hypertension [62–64] that develops secondary to various diseases. Occlusion of the splenic vein results in venous flow draining into collateral veins, including the short gastric vein and left gastroepiploic veins. Increased blood flow in these vessels dilates submucosal veins of the stomach, causing gastric varices that often do not demonstrate clinical symptoms, but can result in hypersplenism or gastrointestinal hemorrhage. 

 Based on their locations, ordinary gastric varices can be classified as either fundal, or cardiac and fundal (located between the cardiac orifice and the fundus); however, in no case of ordinary gastric varices examined using ECDUS do varices show expansion to the curvatura ventriculi major of the gastric body [50]. 

**Figure 5:**
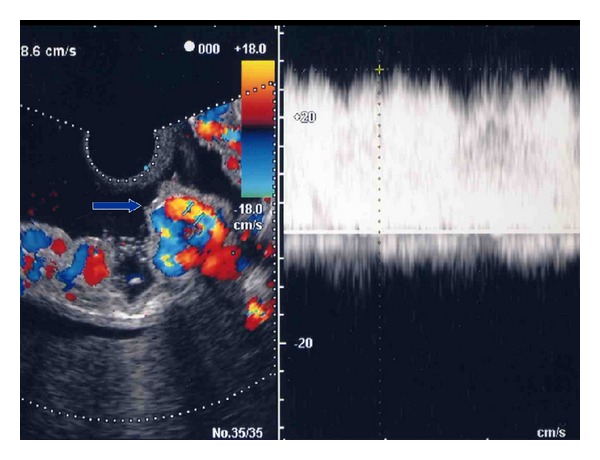
Endoscopic color Doppler ultrasonography of gastric variceal flow clearly depicted a round fundal region at the center, with varices that expanded to the curvatura ventriculi major of the gastric body in splenic vein occlusion.

 Endoscopic evidence is not sufficient to distinguish between gastric varices due to splenic vein occlusion or the gastric fold. Additional images resulting from ECDUS color analysis of gastric variceal flow clearly depicted a round fundal region at the center, with varices that expanded to the curvatura ventriculi major of the gastric body [65] (Figure 5). 

## 8. Summary

- EIS is recommended as the better choice of endoscopic treatments for the anterior branch dominant esophageal varices on hemodynamics of the left gastric vein with ECDUS.
- The direction of blood flow in perforating veins is an important consideration in the therapeutic management of esophageal varices.
- ECDUS can be used to predict the early recurrence of esophageal varices.
- ECDUS is useful for the prediction of gastric variceal bleeding with the measurement of blood flow velocity and wall thickness of submucosal gastric varices.
- ECDUS is a very useful method to evaluate the therapeutic effect of endoscopic treatment with N-butyl-2-cyanoacrylate.

## 9. Conclusions 

 It is important to evaluate the hemodynamics of the portal venous system when treating the esophagogastric varices. ECDUS is a useful modality for the evaluation of the detailed hemodynamics and the therapeutic effects of esophagogastric varices. 

## References

[1] The Veterans Affairs Cooperative Variceal Sclerotherapy Group (1991). Prophylactic sclerotherapy for esophageal varices in men with alcoholic liver disease: a randomized, single-blind, multicenter clinical trial. *The New England Journal of Medicine*.

[2] Goff JS, Reveille RM, Stiegmann GV (1988). Endoscopic sclerotherapy versus endoscopic variceal ligation: esophageal symptoms, complications, and motility. *American Journal of Gastroenterology*.

[3] Trudeau W, Prindiville T (1986). Endoscopic injection sclerosis in bleeding gastric varices. *Gastrointestinal Endoscopy*.

[4] Kanagawa H, Mima S, Kouyama H, Gotoh K, Uchida T, Okuda K (1996). Treatment of gastric fundal varices by balloon-occluded retrograde transvenous obliteration. *Journal of Gastroenterology and Hepatology*.

[5] Watanabe K, Kimura K, Matsutani S, Ohto M, Okuda K (1988). Portal hemodynamics in patients with gastric varices. A study in 230 patients with esophageal and/or gastric varices using portal vein catheterization. *Gastroenterology*.

[6] Hirota S, Matsumoto S, Tomita M, Sako M, Kono M (1999). Retrograde transvenous obliteration of gastric varices. *Radiology*.

[7] Matsumoto A, Hamamoto N, Nomura T (1999). Balloon-occluded retrograde transvenous obliteration of high risk gastric fundal varices. *American Journal of Gastroenterology*.

[8] Kitamoto M, Imamura M, Kamada K (2002). Balloon-occluded retrograde transvenous obliteration of gastric fundal varices with hemorrhage. *American Journal of Roentgenology*.

[9] Ninoi T, Nishida N, Kaminou T (2005). Balloon-occluded retrograde transvenous obliteration of gastric varices with gastrorenal shunt: long-term follow-up in 78 patients. *American Journal of Roentgenology*.

[10] Widrich WC, Srinivasan M, Semine MC, Robbins AH (1984). Collateral pathways of the left gastric vein in portal hypertension. *American Journal of Roentgenology*.

[11] Takashi M, Igarashi M, Hino S (1985). Esophageal varices: correlation of left gastric venography and endoscopy in patients with portal hypertension. *Radiology*.

[12] Hashizume M, Kitano S, Yamaga H, Higashi H, Sugimachi K (1989). Angioarchitectural classification of esophageal varices and paraesophageal veins in selective left gastric venography. *Archives of Surgery*.

[13] Matsutani S, Furuse J, Ishii H, Mizumoto H, Kimura K, Ohto M (1993). Hemodynamics of the left gastric vein in portal hypertension. *Gastroenterology*.

[14] Kegaries DL (1934). The venous plexus of the oesophagus. Its clinical significance. *Surgery, Gynecology & Obstetrics*.

[15] Noda T (1984). Angioarchitectural study of esophageal varices. With special reference to variceal rupture. *Virchows Archiv*.

[16] Arakawa M, Kage M, Okuda K, Benhamou J (1992). The anatomy and pathomorphology of esophageal varices. *Portal Hypertension*.

[17] Hashizume M, Kitano S, Sugimachi K, Sueishi K (1988). Three-dimensional view of the vascular structure of the lower esophagus in clinical portal hypertension. *Hepatology*.

[18] McCormack TT, Rose JD, Smith PM, Johnson AG (1983). Perforating veins and blood flow in oesophageal varices. *The Lancet*.

[19] Vianna A, Hayes PC, Moscoso G (1987). Normal venous circulation of the gastroesophageal junction: a route to understanding varices. *Gastroenterology*.

[20] Hosking SW, Johnson AG (1988). Gastric varices: a proposed classification leading to management. *British Journal of Surgery*.

[21] Sarin SK, Nanda R, Sachdev G (1986). Follow-up of patients after variceal eradication. A comparison of patients with cirrhosis, noncirrhotic portal fibrosis, and extrahepatic obstruction. *Annals of Surgery*.

[22] Beppu K, Inokuchi K, Koyanagi N (1981). Prediction of variceal hemorrhage by esophageal endoscopy. *Gastrointestinal Endoscopy*.

[23] Cho KC, Patel YD, Wachsberg RH, Seeff J (1995). Varices in portal hypertension: evaluation with CT. *Radiographics*.

[24] Sato T, Yamazaki K, Toyota J, Karino Y, Ohmura T, Suga T (1999). Evaluation of magnetic resonance angiography in detection of gastric varices. *Journal of Gastroenterology*.

[25] Balthazar EJ, Megibow A, Naidich D, LeFleur RS (1984). Computed tomographic recognition of gastric varices. *American Journal of Roentgenology*.

[26] Willmann JK, Weishaupt D, Böhm T (2003). Detection of submucosal gastric fundal varices with multi-detector row CT angiography. *Gut*.

[27] Sato T, Yamazaki K, Akaike J (2009). Diagnosis of gastric varices and evaluation of the effectiveness of treatment using transabdominal color Doppler ultrasonography. *Journal of Ultrasound in Medicine*.

[28] Caletti GC, Brocchi E, Baraldini M, Ferrari A, Gibilaro M, Barbara L (1990). Assessment of portal hypertension by endoscopic ultrasonography. *Gastrointestinal Endoscopy*.

[29] Choudhuri G, Dhiman RK, Agarwal DK (1996). Endosonographic evaluation of the venous anatomy around the gastro-esophageal junction in patients with portal hypertension. *Hepato-Gastroenterology*.

[30] Nakamura H, Inoue H, Kawano T, Goseki N, Endo M, Sugihara K (1992). Selection of the treatment for esophagogastric varices—analyses of collateral structures by endoscopic ultrasonography. *Surgical Endoscopy*.

[31] Sato T, Yamazaki K, Toyota J, Karino Y, Ohmura T, Akaike J (2010). Clinical experience with newer electronic radial-type endoscopic color Doppler ultrasonography in the diagnosis of esophageal varices. *Journal of Medical Ultrasonics*.

[32] Sato T, Koito K, Nobuta A (1991). Observation of esophageal varices by endoscopic color Doppler ultrasonography (ECDUS) and usefulness of ECDUS for evaluation of endoscopic injection sclerotherapy. *Gastrointestinal Endoscopy*.

[33] Sato T, Higashino K, Toyota J (1995). Heat-probe coagulation treatment of recurrent intramucosal venous dilatation of the esophagus and endoscopic color Doppler ultrasonographic follow-up. *Digestive Endoscopy*.

[34] Sato T, Yamazaki K, Toyota J, Karino Y, Ohmura T, Suga T (1998). Pulsatile wave in esophageal wall blood vessels after endoscopic therapy for esophageal varices: evaluation by endoscopic color Doppler ultrasonography. *Digestive Endoscopy*.

[35] Hino S, Kakutani H, Ikeda K (2002). Hemodynamic assessment of the left gastric vein in patients with esophageal varices with color Doppler EUS: factors affecting development of esophageal varices. *Gastrointestinal Endoscopy*.

[36] Sato T, Yamazaki K, Toyota J (2003). Visualization of palisade veins in esophageal varices by endoscopic color Doppler ultrasonography. *Digestive Endoscopy*.

[37] Sato T, Yamazaki K, Toyota J (2006). Usefulness of electronic radial endoscopic color Doppler ultrasonography in esophageal varices: comparison with convex type. *Journal of Gastroenterology*.

[38] Yazaki Y, Kawashima T, Sekiya C (1992). F° recurrent esophageal varices—diagnosis, clinical features, and endoscopic injection sclerotherapy for this new type of varices. *Endoscopia Digestiva*.

[39] Sato T, Higashino K, Toyota J (1996). The usefulness of endoscopic color Doppler ultrasonography in the detection of perforating veins of esophageal varices. *Digestive Endoscopy*.

[40] Irisawa A, Obara K, Sakamoto H (1997). The selection and evaluation of the manipulation for endoscopic injection sclerotherapy against esophageal varices with extra esophageal shunt. *Nihon Monmyakuatsu Koshinsho Gakkai Zasshi*.

[41] Saito A, Obara K, Irisawa A (1997). Experience of endoscopic injection sclerotherapy combined with selective endoscopic variceal ligation in 3 patients with esophageal varices accompanied by large extra-esophageal shunt. *Nihon Monmyakuatsu Koshinsho Gakkai Zasshi*.

[42] Inokuchi K, Kobayashi M, Saku M, Nagasue N, Iwaki A, Nakayama S (1977). Characteristics of splanchnic portal circulation in portal hypertension as analyzed by pressure study in clinical cases. *Acta Hepatology*.

[43] Aoki H (1991). The hemodynamics and the treatment of esophago-gastric varices. *Digestive Surgery*.

[44] Reuter SR, Atkin TW (1972). High-dose left gastric angiography for demonstration of esophageal varices. *Radiology*.

[45] Hashizume M, Tanaka K, Inokuchi K (1983). Morphology of gastric microcirculation in cirrhosis. *Hepatology*.

[46] Lunderquist A (1974). Pharmacoangiography of the left gastric artery in oesophageal varices. *Acta Radiologica*.

[47] Kohler B, Riemann JF (1999). The role of endoscopic Doppler-sonography. *Hepato-Gastroenterology*.

[48] Sgouros SN, Bergele C, Avgerinos A (2006). Endoscopic ultrasonography in the diagnosis and management of portal hypertension. Where are we next?. *Digestive and Liver Disease*.

[49] Iwase H, Suga S, Morise K, Kuroiwa A, Yamaguchi T, Horiuchi Y (1995). Color Doppler endoscopic ultrasonography for the evaluation of gastric varices and endoscopic obliteration with cyanoacrylate glue. *Gastrointestinal Endoscopy*.

[50] Sato T, Yamazaki K, Toyota J, Karino Y, Ohmura T, Akaike J (2008). Observation of gastric variceal flow characteristics by endoscopic ultrasonography using color doppler. *American Journal of Gastroenterology*.

[51] Suzuki T, Matsutani S, Umebara K (2000). EUS changes predictive for recurrence of esophageal varices in patients treated by combined endoscopic ligation and sclerotherapy. *Gastrointestinal Endoscopy*.

[52] Konishi Y, Nakamura T, Kida H, Seno H, Okazaki K, Chiba T (2002). Catheter US probe EUS evaluation of gastric cardia and perigastric vascular structures to predict esophageal variceal recurrence. *Gastrointestinal Endoscopy*.

[53] Seno H, Konishi Y, Wada M, Fukui H, Okazaki K, Chiba T (2006). Endoscopic ultrasonograph evaluation of vascular structures in the gastric cardia predicts esophageal variceal recurrence following endoscopic treatment. *Journal of Gastroenterology and Hepatology*.

[54] Irisawa A, Saito A, Obara K (2001). Endoscopic recurrence of esophageal varices is associated with the specific EUS abnormalities: Severe peri-esophageal collateral veins and large perforating veins. *Gastrointestinal Endoscopy*.

[55] Shibukawa G, Irisawa A, Saito A (2004). Variceal recurrence after endoscopic sclerotherapy associated with the perforating veins in lower esophagus independently. *Hepato-Gastroenterology*.

[56] Sakai T, Iwao T, Oho K, Toyonaga A, Tanikawa K (1997). Influence of extravariceal collateral channel pattern on recurrence of esophageal varices after sclerotherapy. *Journal of Gastroenterology*.

[57] Dilawari JB, Raju GS, Chawla YK (1989). Development of large spleno-adreno-renal shunt after endoscopic sclerotherapy. *Gastroenterology*.

[58] Ito K, Matsutani S, Maruyama H (2006). Study of hemodynamic changes in portal systemic shunts and their relation to variceal relapse after endoscopic variceal ligation combined with ethanol sclerotherapy. *Journal of Gastroenterology*.

[59] Sato T, Yamazaki K, Toyota J, Karino Y, Ohmura T, Akaike J (2009). Endoscopic ultrasonographic evaluation of hemodynamics related to variceal relapse in esophageal variceal patients. *Hepatology Research*.

[60] Soehendra N, Grimm H, Nam VC, Berger B (1987). N-butyl-2-cyanoacrylate: a supplement to endoscopic sclerotherapy. *Endoscopy*.

[61] Sato T (2010). Endoscopic ultrasonographic findings before and after sclerotherapy for gastric varices. *Journal of Medical Ultrasonics*.

[62] Sutton JP, Yarborough DY, Richards JT (1970). Isolated splenic vein occlusion. Review of literature and report of an additional case. *Archives of Surgery*.

[63] Babb RR (1976). Splenic vein obstruction: a curable cause of variceal bleeding. *American Journal of Digestive Diseases*.

[64] Muhletaler C, Gerlock AJ, Goncharenko V (1979). Gastric varices secondary to splenic vein occlusion: radiographic diagnosis and clinical significance. *Radiology*.

[65] Sato T (2012). Gastric varices secondary to splenic vein occlusion: endoscopic color Doppler ultrasonography aids diagnosis. *Journal of Medical Ultrasonics*.

